# Evidence of Cooperative Effects for the Fe(phen)_2_(NCS)_2_ Spin Crossover Molecular Complex in Polyaniline Plus Iron Magnetite

**DOI:** 10.3390/molecules29194574

**Published:** 2024-09-26

**Authors:** Wai Kiat Chin, Binny Tamang, M. Zaid Zaz, Arjun Subedi, Gauthami Viswan, Alpha T. N’Diaye, Rebecca Y. Lai, Peter A. Dowben

**Affiliations:** 1 Department of Physics and Astronomy, University of Nebraska-Lincoln, Lincoln, NE 68588, USA; wchin6@huskers.unl.edu (W.K.C.); zzaz2@huskers.unl.edu (M.Z.Z.); arjun.subedi@huskers.unl.edu (A.S.); gviswan2@huskers.unl.edu (G.V.); 2 Department of Chemistry, University of Nebraska-Lincoln, Lincoln, NE 68588, USA; btamang2@huskers.unl.edu; 3 Advanced Light Source, Lawrence Berkeley National Laboratory, Berkeley, CA 94720, USA; atndiaye@lbl.gov

**Keywords:** spin crossover composites, cooperative effects, molecular-based memory device, functional materials

## Abstract

The spin crossover complex Fe(phen)_2_(NCS)_2_ and its composite, Fe(phen)_2_(NCS)_2_, combined with the conducting polymer polyaniline (PANI) plus varying concentrations of iron magnetite (Fe_3_O_4_) nanoparticles were studied. A cooperative effect is evident from the hysteresis width in the plot of magnetic susceptibility multiplied by temperature versus temperature ($\chi_{m}T$ versus T) for Fe(phen)_2_(NCS)_2_ with PANI plus varying concentrations of Fe_3_O_4_ nanoparticles. The hysteresis width in the composites vary no more than 2 K with respect to the pristine Fe(phen)_2_(NCS)_2_ spin crossover crystallites despite the fact that there exists a high degree of miscibility of the Fe(phen)_2_(NCS)_2_ spin crossover complex with the PANI. The Fe_3_O_4_ nanoparticles in the Fe(phen)_2_(NCS)_2_ plus PANI composite tend to agglomerate at higher concentrations regardless of the spin state of Fe(phen)_2_(NCS)_2_. Of note is that the Fe_3_O_4_ nanoparticles are shown to be antiferromagnetically coupled with the Fe(phen)_2_(NCS)_2_ when Fe(phen)_2_(NCS)_2_ is in the high spin state.

## 1. Introduction

The study of the effects of mixing a conducting polymer with bi-stable spin crossover (SCO) molecular complexes is fairly recent and sparse [1,2,3,4,5,6,7,8,9]. Efforts have been made in an attempt to lower the on-state resistance of spin crossover molecular films by forming composites with various conducting polymers [1,3,4,6], largely with the goal of improving the utility of SCO film-based devices [4,10,11,12,13,14,15,16,17,18,19,20]. A previous study of a polymeric spin crossover [Fe(Htrz)_2_(trz)](BF)_4_ plus the conducting polymer polyaniline (PANI) showed a reduction in the hysteresis width in the $\chi_{m}T$ versus T, which is an indication of a reduced cooperative effect [5]. However, the cooperative effect of [Fe(Htrz)_2_(trz)](BF)_4_ was restored and enhanced following the addition of iron magnetite (Fe_3_O_4_) nanoparticles into the [Fe(Htrz)_2_(trz)](BF)_4_ plus PANI composite. This was understood to be due to the phase separation of [Fe(Htrz)_2_(trz)](BF)_4_ in the PANI matrix caused by the addition of Fe_3_O_4_ nanoparticles. Such a phase separation turned the miscible [Fe(Htrz)_2_(trz)](BF)_4_ plus PANI composite into immiscible domains of [Fe(Htrz)_2_(trz)](BF)_4_ and PANI, where the Fe_3_O_4_ nanoparticles are preferentially segregated to the [Fe(Htrz)_2_(trz)](BF)_4_ domains. Overall, the prior study has suggested that the miscibility gap in the SCO-polymer system could be easily perturbed.

Similarly, the spin crossover complex Fe(phen)_2_(NCS)_2_ has been much studied [21,22,23,24,25,26,27,28,29,30,31,32,33,34,35,36,37,38,39,40,41], and the spin state transition temperature for this complex is in the vicinity of 176 K [27,28,29,30,31,32,33,34,37,38,39]. Prior studies of Mn-doped and Zn-doped Fe(phen)_2_(NCS)_2_ thin films [38,39,40], as well as [Fe(phen)_2_(NCS)_2_] crystallites coated with an organic, like glycerol [41], showed that the hysteresis width, in $\chi_{m}T$ versus T, varied depending on the dopant [38,39] and the coating layer [41], respectively. In this study, we observed that for Fe(phen)_2_(NCS)_2_ plus PANI plus varying concentrations of Fe_3_O_4_ nanoparticle composites, the hysteresis width of $\chi_{m}T$ versus T remains largely unchanged. The hysteresis width behavior of Fe(phen)_2_(NCS)_2_ plus PANI plus varying concentrations of Fe_3_O_4_ nanoparticles is starkly different from that of the [Fe(Htrz)_2_(trz)](BF)_4_ plus PANI plus Fe_3_O_4_ nanoparticles (1% loading by weight) [5], indicating that the dispersion of a spin crossover complex in PANI and the exchange between the spin crossover complex and the Fe_3_O_4_ nanoparticles, which is mediated by PANI, depends very much on the choice of spin crossover molecule. With this work, there are now examples of both ferromagnetic [5] and antiferromagnetic coupling between a spin crossover molecular complex and iron magnetite nanoparticles, mediated by polyaniline.

## 2. Experimental Details

All the reagents and solvents were used as received. Ammonium iron (II) sulfate hexahydrate (99%), aniline (99.8%), ammonium persulfate (APS), and hydrochloric acid (HCl) (ACS reagent, 37%) were purchased from Fisher Scientific (Hampton, NH, USA). 1–10 phenanthroline monohydrate was purchased from Chem-Impex (Wood Dale, IL, USA). Sodium thiocyanate (NaSCN) (99%) was purchased from Avantor (Radnor Township, PA, USA). Ethanol and methanol (≥99.9%) were purchased from Aldrich (St. Louis, MO, USA). Iron (II, III) nano-powders (50–100 nm particle size, 97% trace metals) were purchased from Millipore Sigma (Burlington, MA, USA). The solutions used in this study were made with deionized (DI) water purified through a Millipore Synergy system (18.2 MΩ·cm, Millipore, Billerica, MA, USA).

The method used for the synthesis of Fe(phen)_2_(NCS)_2_ was adapted from the one used by Akabori et al. [42]. In brief, 1.95 g of ammonium iron (II) sulfate hexahydrate and 3.00 g of 1–10 phenanthroline were dissolved together in 200 mL of DI water, resulting in a dark-red solution. Next, 21 g of NaSCN was added to 15 mL of DI water to create a saturated solution, and this solution was added to the dark-red solution while stirring. Fine crystals were observed immediately, but the solution mixture was stirred for another four hours at room temperature to complete the reaction. The crude product was collected on Whatman filter paper and rinsed with methanol. It was further purified via recrystallization in methanol and dried in air. The purity was determined by IR spectroscopy (Supplementary Materials Figure S1).

The synthesis of PANI was adapted from the methods used by Tang et al. [43] and Adams et al. [44]. A solution containing 0.25 M aniline (683 µL) in 30 mL of DI water was placed in an ice bath with magnetic stirring at 800 rpm, and 0.25 M APS in 10 mL of 1 M HCl was added to the aniline solution. The solution was allowed to stir for six hours at 0 °C. A dark green precipitate began to form within the first half hour. After six hours, the reaction was left to proceed overnight with stirring at room temperature. The dark green precipitate was collected using ultracentrifugation at 4500 rpm for 30 min and washed several times with 1 M HCl, ethanol, and DI water.

The fabrication of the composite thin films of Fe(phen)_2_(NCS)_2_ plus PANI and Fe(phen)_2_(NCS)_2_ together with PANI and Fe_3_O_4_ nanoparticles was accomplished through a solvent approach. In total, 20 mg of Fe(phen)_2_(NCS)_2_ and 20 mg of PANI were added to 5 mL of methanol, and this mixture was sonicated in a Branson 2510R-MT bath sonicator at room temperature for 10 min to create a uniform dispersion. This mixture, also known as the 1:1 Fe(phen)_2_(NCS)_2_/PANI bi-composite, was then dried to completion in air prior to vibrating sample magnetometry (VSM) analysis. The 1:1 Fe(phen)_2_(NCS)_2_/PANI bi-composite ratio is emphasized in this work to make the comparison with the prior study of the [Fe(Htrz)_2_(trz)](BF)_4_ plus PANI composite [5] more facile. To fabricate the tri-composite samples containing Fe(phen)_2_(NCS)_2_, PANI, and Fe_3_O_4_, different amounts (1, 5, and 10% by weight) of Fe_3_O_4_ nanoparticles were added to the 1:1 Fe(phen)_2_(NCS)_2_/PANI bi-composite and sonicated again for 10 min until uniformly dispersed. These samples were dried in air prior to VSM analysis.

The measurements of DC magnetic susceptibility times temperature ($\chi_{m}T$) were carried out in DynaCool PPMS from Quantum Design. The elemental mapping of iron and sulfur in the [Fe(phen)_2_(NCS)_2_] and its composites was performed using the FEI Tecnai Osiris S/TEM with the means of energy-dispersive X-ray spectroscopy (EDAX) and high-angle annular dark-field imaging mode with a 200 kV accelerating voltage of scanning transmission electron microscopy (HAADF-STEM). The Fe(phen)_2_(NCS)_2_ and its composites plus polyaniline (PANI) plus various concentrations of Fe_3_O_4_ nanoparticle solutions were drop-casted on formvar/carbon 200 mesh copper TEM grids from Ted Pella.

The room temperature DC current-voltage I(V) measurements of Fe(phen)_2_(NCS)_2_ plus PANI plus Fe_3_O_4_ (10% loading by weight) drop-cast between two copper electrodes were taken using the two-point probe method and recorded using a 4200A SCS parameter analyzer connected to a Lakeshore cryogenic probe station. The measurements were carried out in the absence of visible light illumination and a sweep rate of 260 ms per data point.

The coupling between the Fe(phen)_2_(NCS)_2_ and Fe_3_O_4_ nanoparticles in the composite was probed with X-ray absorption spectroscopy (XAS) and X-ray magnetic circular dichroism spectroscopy (XMCD), as a function of temperature, at the Advanced Light Source (Lawrence Berkeley National Laboratory) beamline 6.3.1, with a constant circularly polarized light whose estimated degree of polarization was 0.66. Throughout the experiments, the XAS spectra were taken in the total electron yield mode across the Fe $L_{3,2}$ edges ranging from 700 eV to 735 eV with an alternating magnetic field of ±1.5 T, which are anti-parallel ($\sigma^{↓}$) and parallel ($\sigma^{↑}$) with respect to the helicity of the incoming photon. The XMCD signal was later evaluated as a difference of the two XAS spectra taken with two oppositely applied magnetic fields.

## 3. Miscibility and Cooperativity

**Figure 1:**
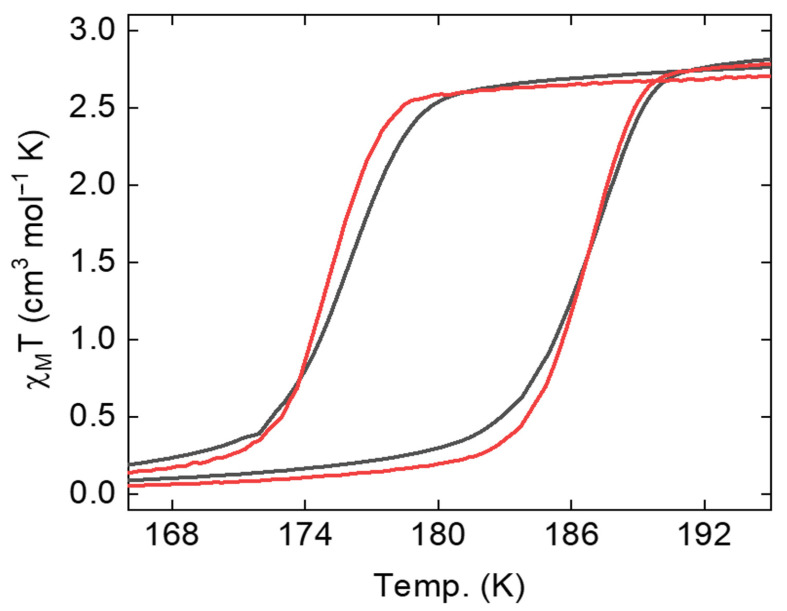
The enlarged view of the magnetic susceptibility time temperature, $\chi_{M}T$, versus T plot for Fe(phen)_2_(NCS)_2_ crystallites (gray) and its composite Fe(phen)_2_(NCS)_2_ plus PANI (1:1 Fe(phen)_2_(NCS)_2_/PANI weight %) plotted in red.

The intermolecular interactions, namely the cooperative effects [45,46,47,48], were examined through thermal hysteresis in terms of the magnetic susceptibility ($\chi_{m}$) times temperature versus temperature, i.e., the hysteresis width in the plot of $\chi_{m}T$ versus T. The hysteresis width, in principle, is a measure of the strength of the cooperativity between adjacent SCO molecules, with a larger hysteresis width equivalent to stronger cooperative effects [45,46,47,48]. The hysteresis widths in the $\chi_{m}T$ versus T for Fe(phen)_2_(NCS)_2_ and for the combination of Fe(phen)_2_(NCS)_2_ plus PANI were 10.6 K and 11.3 K, respectively, as shown in Figure 1 (Supplementary Figure S4). The percentage error in the measured $\chi_{m}T$ versus T hysteresis width was less than 6%. The observed hysteresis width in this study is significantly larger than the 0.15 to 1.5 K [27,29,30,33,34] and 6.1–8.5 K [31] reported previously but roughly similar to the hysteresis width of Mn-doped and Zn-doped Fe(phen)_2_(NCS)_2_ thin films, which were 10 K [39] and 14 K [38]. This is surprising as this is very different from the example of [Fe(Htrz)_2_(trz)](BF)_4_, where a significant 7 K reduction of $\chi_{m}T$ versus T hysteresis width was observed upon mixing with PANI [5].

**Figure 2:**
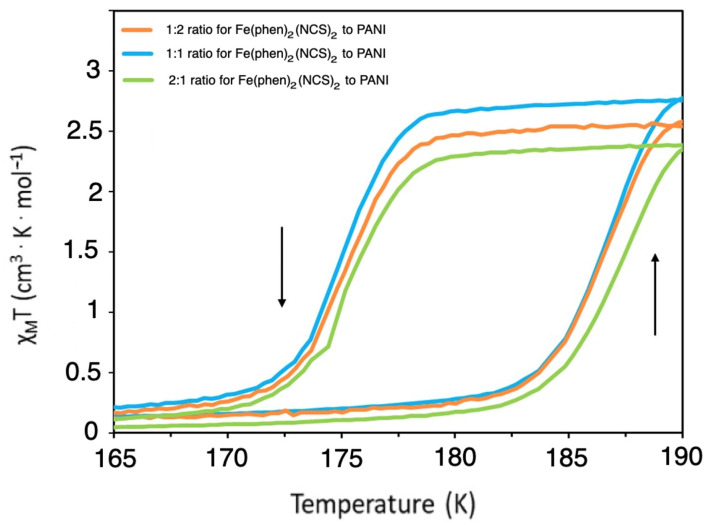
The thermal hysteresis behavior of $\chi_{M}T$ versus T for Fe(phen)_2_(NCS)_2_ to PANI bi-composites in the different ratios of 2:1 (green), 1:1 (blue), and 1:2 (orange). The arrows indicate increasing (up) and decreasing (down) temperature paths.

A comparison of the mixtures of Fe(phen)_2_(NCS)_2_ with PANI in the ratios of 1:2, 1:1, and 2:1, as seen in Figure 2, illustrates that the magnetization for the Fe(phen)_2_(NCS)_2_ to PANI in the ratio of 1:1 is slightly larger than that seen for the ratios of 1:2 and 2:1 Fe(phen)_2_(NCS)_2_ to PANI compositions. The temperature-dependent hysteresis varies only slightly, with the hysteresis loop (ΔT_1/2_) for both the 1:1 Fe(phen)_2_(NCS)_2_ to PANI and the 1:2 Fe(phen)_2_(NCS)_2_ to PANI bi-composite measuring ~11 K, but for the 2:1 Fe(phen)_2_(NCS)_2_ to PANI bi-composite, the hysteresis is ~12 K.

**Figure 3:**
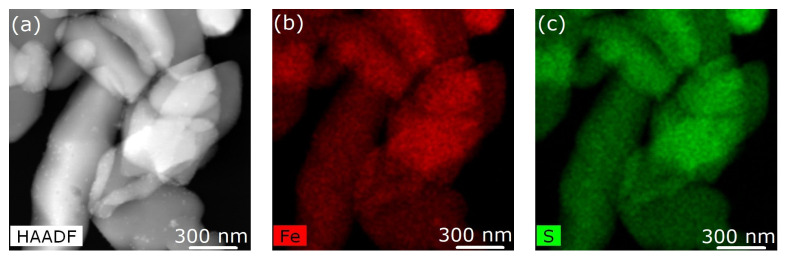
(**a**) Image of pristine Fe(phen)_2_(NCS)_2_ obtained using scanning transmission microscopy in high-angle annular dark-field mode. (**b**,**c**) Elemental mapping of iron and sulfur of the [Fe(phen)_2_(NCS)_2_], respectively.

**Figure 4:**
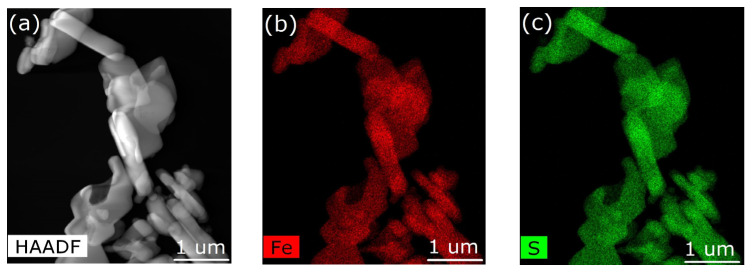
(**a**) Image of Fe(phen)_2_(NCS)_2_ combined with polyaniline (PANI) obtained using scanning transmission microscopy in high-angle annular dark-field mode. (**b**,**c**) Elemental mapping of iron and sulfur of the [Fe(phen)_2_(NCS)_2_], respectively.

From the elemental mapping of both iron and sulfur of the [Fe(phen)_2_(NCS)_2_] in the pristine Fe(phen)_2_(NCS)_2_ crystallites and the Fe(phen)_2_(NCS)_2_ plus PANI composite, respectively, in Figure 3 and Figure 4, the fact that the Fe(phen)_2_(NCS)_2_ is uniformly distributed within the PANI matrix is clear. In other words, there is a high degree of miscibility of Fe(phen)_2_(NCS)_2_ within the PANI matrix. The two different spin crossover complexes, [Fe(Htrz)_2_(trz)](BF)_4_ and Fe(phen)_2_(NCS)_2_, while both are apparently miscible with the PANI, do not mix the same way in the PANI, judging from the differences in the changes to the hysteresis width in the $\chi_{m}T$ versus T seen with mixing [Fe(Htrz)_2_(trz)](BF)_4_ with PANI and not seen when Fe(phen)_2_(NCS)_2_ was mixed with PANI, as noted above. One notable difference between these two spin crossover molecules is that [Fe(Htrz)_2_(trz)](BF)_4_ is a polymeric spin crossover complex and Fe(phen)_2_(NCS)_2_ is not.

We offer two possible explanations as to why the cooperative effects (as indicated by the temperature-dependent hysteresis of the susceptibility) of Fe(phen)_2_(NCS)_2_ moieties mixed in the PANI remain largely unchanged. First, there could exist tiny Fe(phen)_2_(NCS)_2_ crystallites with the correct packing for enhancing the hysteresis [31] that are evenly distributed within the PANI matrix. For Fe(phen)_2_(NCS)_2_, the hysteresis width can also be affected by the crystallite quality [31,34]. Alternatively, there is the possibility of strong cooperativity between Fe(phen)_2_(NCS)_2_ moieties in widely distributed crystallites or PANI, which enhances the $\chi_{m}T$ versus T hysteresis observed in this study here. The large and unperturbed $\chi_{m}T$ versus T hysteresis suggests that PANI encapsulates the Fe(phen)_2_(NCS)_2_, which could enhance the overall cooperativity. Similar polymer-encapsulation-induced hysteresis enhancement of [Fe(phen)_2_(NCS)_2_] has been reported [25,41]. The explanation is consistent with the observations where there is strong cooperativity for Fe(phen)_2_(NCS)_2_ when encapsulated by organics [7,9,25,41]. The latter explanation is consistent with the observed increased hysteretic spin transition behavior of Fe(phen)_2_(NCS)_2_ in several different embedding matrices [9].

## 4. Phase Separation and Cooperativity

**Figure 5:**
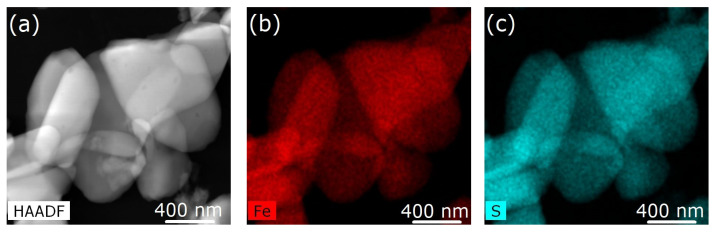
(**a**) Image of Fe(phen)_2_(NCS)_2_ plus PANI plus Fe_3_O_4_ nanoparticles (1% loading by weight) obtained using scanning transmission microscopy in high-angle annular dark-field mode. (**b**,**c**) Elemental mapping of iron and sulfur of the [Fe(phen)_2_(NCS)_2_], respectively.

**Figure 6:**
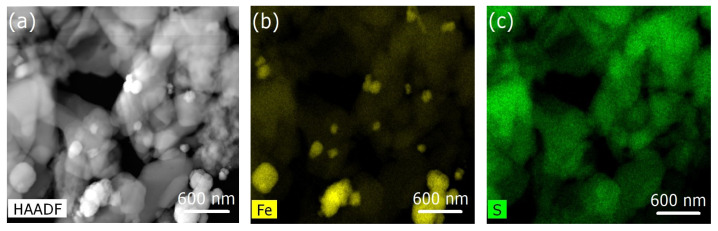
(**a**) Image of Fe(phen)_2_(NCS)_2_ plus PANI plus Fe_3_O_4_ nanoparticles (5% loading by weight) obtained using scanning transmission microscopy in high-angle annular dark-field mode. (**b**,**c**) Elemental mapping of iron and sulfur of the [Fe(phen)_2_(NCS)_2_], respectively.

**Figure 7:**
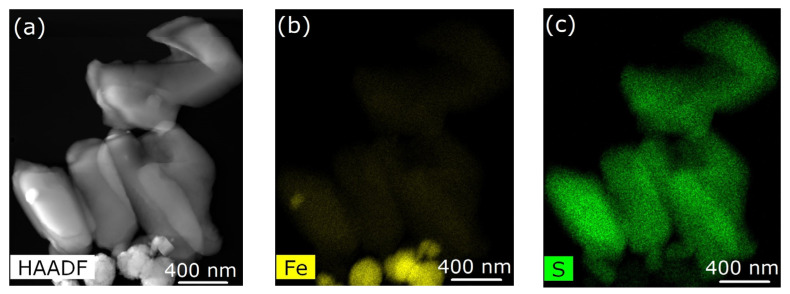
(**a**) Image of Fe(phen)_2_(NCS)_2_ plus PANI plus Fe_3_O_4_ nanoparticles (10% loading by weight) obtained using scanning transmission microscopy in high-angle annular dark-field mode. (**b**,**c**) Elemental mapping of iron and sulfur of the [Fe(phen)_2_(NCS)_2_], respectively.

With increasing Fe_3_O_4_ nanoparticle concentrations in the Fe(phen)_2_(NCS)_2_ plus PANI composite, the Fe_3_O_4_ nanoparticles tend to agglomerate and become less uniformly distributed (Figure 5b, Figure 6b and Figure 7b) in the PANI matrix, while the Fe(phen)_2_(NCS)_2_ remains uniformly distributed within the PANI, as indicated by the sulfur spatial map (Figure 5c, Figure 6c and Figure 7c). Although there is always a possibility that the coupling between the Fe_3_O_4_ nanoparticles could lead to magnetic frustration, this is excluded here as the overall paramagnetic susceptibility scales with the Fe_3_O_4_ nanoparticle concentration in the Fe(phen)_2_(NCS)_2_ plus PANI composite.

**Figure 8:**
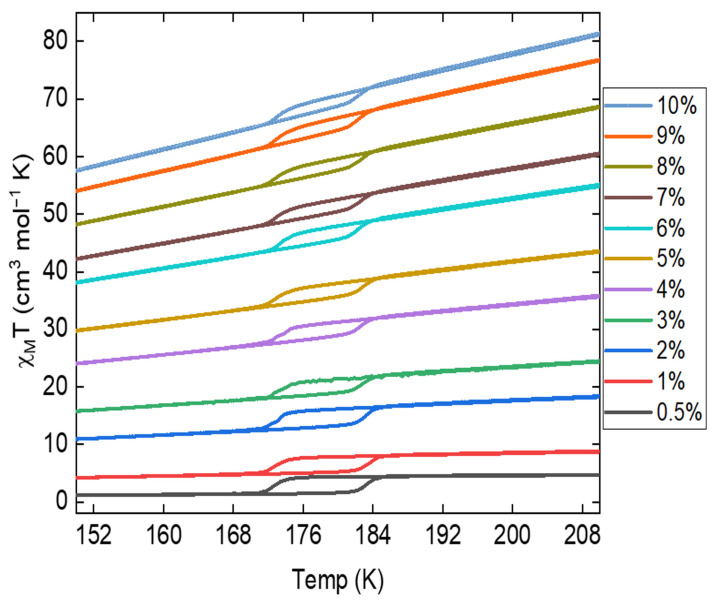
The $\chi_{M}T$ versus T plots for composites of Fe(phen)_2_(NCS)_2_ plus PANI plus varying concentrations of Fe_3_O_4_ (percent loading, by weight), with an applied magnetic field of 2 T.

As shown in Figure 8, the addition of Fe_3_O_4_ nanoparticles to the Fe(phen)_2_(NCS)_2_ plus PANI composite leads to an increasing paramagnetic susceptibility background with an increasing concentration of Fe_3_O_4_ nanoparticles in the Fe(phen)_2_(NCS)_2_ plus PANI composite. In general, as shown in Figure 8, the paramagnetic susceptibility at a fixed temperature is linearly proportional to the concentration of Fe_3_O_4_ nanoparticles in the Fe(phen)_2_(NCS)_2_ plus PANI composite.

The hysteresis width in the $\chi_{m}T$ versus T remains remarkably consistent, with changes no more than 2 K across varying concentrations of Fe_3_O_4_ nanoparticles in the Fe(phen)_2_(NCS)_2_ plus PANI composite (Figure 8, Supplementary Materials Table S1, Figures S2 and S3). These largely consistent hysteresis widths indicate that the cooperative effect seen for Fe(phen)_2_(NCS)_2_ is stable and remains unperturbed upon mixing with the PANI and the addition of Fe_3_O_4_ nanoparticles into the composite. This differs from the doped Fe(phen)_2_(NCS)_2_ thin film [38,39], where Mn doping has a profound effect on the hysteresis width in the temperature.

**Figure 9:**
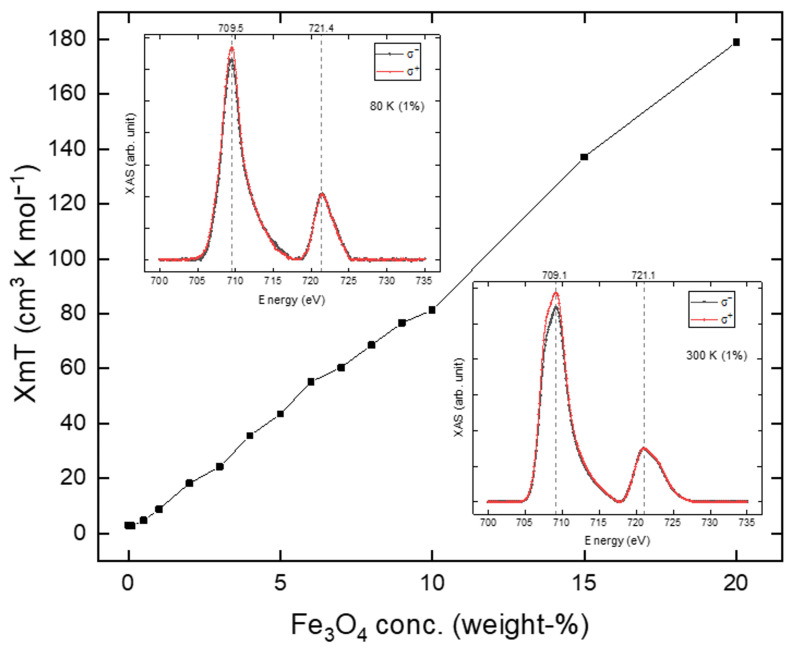
The value $\chi_{M}T$ is plotted as a function of the Fe_3_O_4_ nanoparticle concentration in Fe(phen)_2_(NCS)_2_ plus PANI composite, for T = 210 K. Insets are the X-ray absorption spectra of Fe L_3_ (2p_3/2_) and L_2_ (2p_1/2_) edges at 80 K (top left corner) and 300 K (bottom right corner) of the Fe(phen)_2_(NCS)_2_ plus PANI plus Fe_3_O_4_ (1% loading by weight).

**Figure 10:**
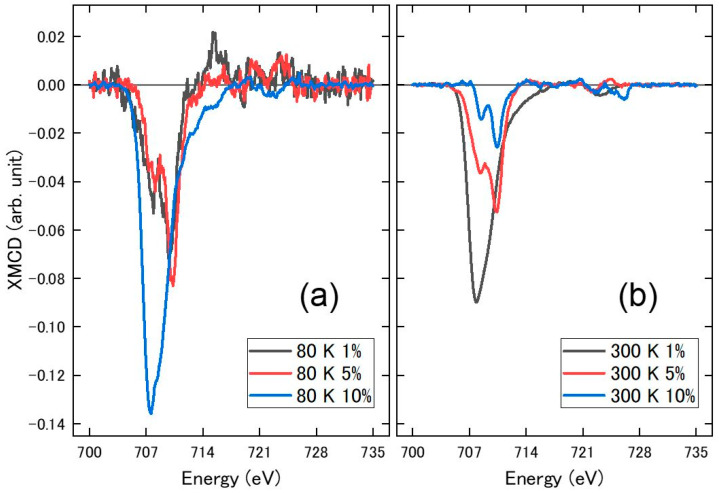
The Fe ($L_{3}$ and $L_{2}$ edges) XMCD signals for Fe(phen)_2_(NCS)_2_ plus PANI plus Fe_3_O_4_ nanoparticles (1%, 5% and 10% loading by weight). Data were taken for Fe(phen)_2_(NCS)_2_ in the low spin state (**a**) at 80 K and Fe(phen)_2_(NCS)_2_ in the high spin state (**b**) at 300 K.

Above the spin state transition temperature, Fe(phen)_2_(NCS)_2_ has a significant magnetic moment that can overwhelm the moment from the Fe_3_O_4_ nanoparticles at very low Fe_3_O_4_ nanoparticle concentrations, as shown in the insets in Figure 9 and Figure 10. For the Fe(phen)_2_(NCS)_2_ plus PANI plus Fe_3_O_4_ nanoparticle (1% loading by weight) composite, there is an increase in the XMCD signal at the Fe 2p core edges (Figure 10) as the Fe(phen)_2_(NCS)_2_ goes from the diamagnetic low spin state (at 80 K) to the paramagnetic high spin state (at 300 K). At higher Fe_3_O_4_ nanoparticle concentrations in the Fe(phen)_2_(NCS)_2_ plus PANI composite, the effect of the Fe(phen)_2_(NCS)_2_ spin state is more complex.

**Figure 11:**
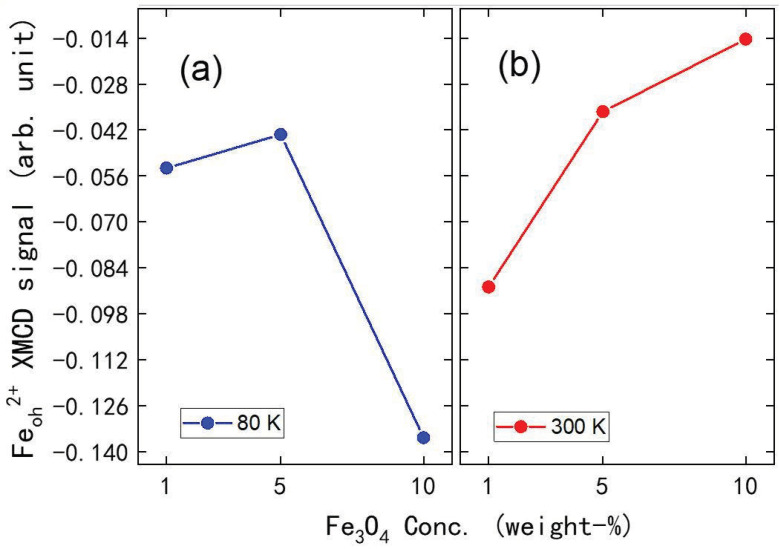
The XMCD signal of the ${Fe}_{Oh}^{2+}$ site for varying concentrations (% by weight) of Fe_3_O_4_ nanoparticles in the Fe(phen)_2_(NCS)_2_ plus PANI composite at (**a**) 80 K and (**b**) 300 K.

The XMCD response, Figure 10a, extracted from the XAS spectra (the insets in Figure 9 and Supplementary Materials Figures S5–S8) with oppositely applied magnetic fields does increase with increasing Fe_3_O_4_ nanoparticle concentrations when Fe(phen)_2_(NCS)_2_ is in the diamagnetic low spin state (at 80 K). The increase in the XMCD signal is not linear with respect to the increasing Fe_3_O_4_ nanoparticle concentration (Figure 11a). This is true even for Fe_3_O_4_ nanoparticles in the Fe(phen)_2_(NCS)_2_ plus PANI composite at 80 K, where Fe(phen)_2_(NCS)_2_ is in a low spin state (diamagnetic) and does not contribute to the net magnetization. The surmise is that the Fe_3_O_4_ nanoparticles are the primary overall XMCD signal source when Fe(phen)_2_(NCS)_2_ is in a low spin state. This hypothesis that the Fe_3_O_4_ nanoparticles are the primary overall XMCD signal source when Fe(phen)_2_(NCS)_2_ is in a low spin state is consistent with the fact that below the spin crossover transition temperature (<172 K), the magnetic susceptibility is fairly constant in temperature. The non-linear increase in the XMCD signal with respect to the increasing concentrations of Fe_3_O_4_ nanoparticles can be attributed to the fact that the agglomeration of Fe_3_O_4_ nanoparticles (Figure 4b, Figure 5b and Figure 6b) is ferromagnetically coupled with the Fe(phen)_2_(NCS)_2_ in the Fe(phen)_2_(NCS)_2_ plus PANI composite, as the Fe_3_O_4_ nanoparticles aggregate.

Despite the fact that the magnetic susceptibility times temperature, $\chi_{M}T$, at a constant temperature of 210 K increases linearly with respect to an increasing concentration of the Fe_3_O_4_ nanoparticles, as shown in Figure 9, a comparison of the XMCD signal at the Fe L_3_ (2p_3/2_) edge in Figure 10b provides evidence that there is anti-ferromagnetic coupling between the Fe_3_O_4_ nanoparticles and the Fe(phen)_2_(NCS)_2_ in the Fe(phen)_2_(NCS)_2_ plus PANI plus Fe_3_O_4_ nanoparticles composite. Hence, there is an overall reduction in the XMCD signal (Figure 10b, Supplementary Figure S8), with an increasing concentration of Fe_3_O_4_ nanoparticles in the Fe(phen)_2_(NCS)_2_ plus PANI composite when the [Fe(phen)_2_(NCS)_2_] is in a high spin state. The decrease in the XMCD response is rather significant, and, therefore, the antiferromagnetic coupling between both the Fe(phen)_2_(NCS)_2_ and the Fe_3_O_4_ nanoparticles cannot be restricted to only the Fe(phen)_2_(NCS)_2_ at the surface of the Fe_3_O_4_ nanoparticles. Furthermore, it implies that Fe(phen)_2_(NCS)_2_ retains nontrivial paramagnetic correlation lengths in PANI.

## 5. I(V) Characteristics

**Figure 12:**
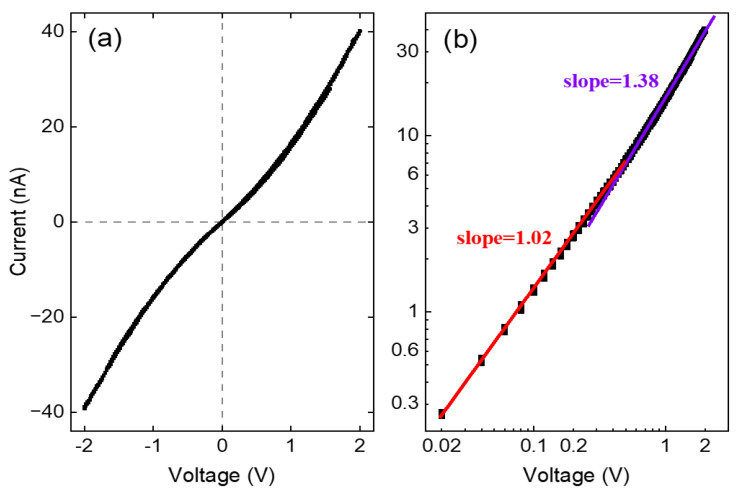
(**a**) The dc I(V) characteristics of Fe(phen)_2_(NCS)_2_ plus PANI plus Fe_3_O_4_ (10% by weight) and (**b**) double-log plot of the I(V) characteristics.

The transport measurements also suggest that the behavior of Fe(phen)_2_(NCS)_2_ in the composites of Fe(phen)_2_(NCS)_2_ plus PANI plus Fe_3_O_4_ is not perturbed by the addition of Fe_3_O_4_ nanoparticles. The room temperature DC current-voltage I(V) measurement shown in Figure 12 is, in fact, characteristic of the transport behavior of Fe(phen)_2_(NCS)_2_ by itself [21], which was carried out on thin films of Fe(phen)_2_(NCS)_2_ plus PANI plus Fe_3_O_4_ (10% loading by weight) drop-casted between two copper electrodes on a glass substrate without light illumination. As anticipated from the transport behavior of Fe(phen)_2_(NCS)_2_ by itself [21], the transport characteristic is Ohmic with a linear slope of 1.02, as plotted in the double log I(V) curve, Figure 12b, approximately below 0.6 V. Above 0.6 V, the transport characteristic remains linear with a slope of 1.38, which is an indication that it tends towards a space charge-limited current regime. Such space charge-limited current behavior of the Fe(phen)_2_(NCS)_2_ thin film itself has been reported previously with a linear slope of 2.04 [21]. The slope of 1.38 obtained in this study can be understood as a diminishing of the space charge with Fe(phen)_2_(NCS)_2_ in the PANI matrix. This further confirms that mixing Fe(phen)_2_(NCS)_2_ with the PANI, which is nominally a conducting polymer, reduces the overall on-state resistance.

We can understand part of this overall behavior, i.e., the diminishing of the space charge, when different concentrations of Fe_3_O_4_ nanoparticles are added to the Fe(phen)_2_(NCS)_2_ plus PANI composite by noting that Fe(phen)_2_(NCS)_2_ remains uniformly mixed with PANI, at least on the scale of the scanning TEM, but as the concentration of Fe_3_O_4_ nanoparticles increases, the Fe_3_O_4_ nanoparticles are phase separated, as seen in Figure 4, Figure 5 and Figure 6.

## 6. Summary

The cooperative effect, in general, is sensitive to the sample synthesis method [9,29,31,34,42]. Here, the hysteretic behavior of Fe(phen)_2_(NCS)_2_ is larger than generally observed. Yet the large hysteretic behavior of approximately 10 to 11 K is largely retained upon the addition of PANI and Fe_3_O_4_ nanoparticles, unlike what was observed with [Fe(Htrz)_2_(trz)](BF)_4_ combined with PANI [5] and Mn-doped Fe(phen)_2_(NCS)_2_ [38,39]. At the same time, the cooperative effect in this study is among the largest of those reported in the literature [27,28,29,30,31,32,33,34], although it is smaller than the 14 K inferred from [38]. While small groupings of Fe(phen)_2_(NCS)_2_ moieties may occur in the PANI matrix, there is no phase separation of the Fe(phen)_2_(NCS)_2_ from the PANI. In other words, the Fe(phen)_2_(NCS)_2_ tends to mix fairly homogeneously with the PANI.

The agglomeration of Fe_3_O_4_ nanoparticles, which is an indication of the phase separation of Fe_3_O_4_ nanoparticles in the Fe(phen)_2_(NCS)_2_ plus PANI composite, is observed and it is observed to be directly proportional to the concentration of Fe_3_O_4_ nanoparticles. However, the Fe(phen)_2_(NCS)_2_ spin crossover molecules remain uniformly distributed within the PANI in all cases, which suggests that there exists a relatively high degree of miscibility.

Surprisingly, adding varying concentrations of Fe_3_O_4_ nanoparticles into the Fe(phen)_2_(NCS)_2_ plus PANI composite did not perturb the cooperativity. The hysteresis width in the $\chi_{m}T$ versus T for Fe(phen)_2_(NCS)_2_, at the spin state transition, remains consistent, with a hysteresis width change of less than 2 K throughout various concentrations of Fe_3_O_4_ nanoparticles in the Fe(phen)_2_(NCS)_2_ plus PANI composite. In addition, from the XMCD at the Fe edges, we observed that the overall XMCD signal does not increase in a linear fashion with respect to an increasing concentration of Fe_3_O_4_ nanoparticles in the Fe(phen)_2_(NCS)_2_ plus PANI composite. In addition, the phase-separated Fe_3_O_4_ nanoparticles in the Fe(phen)_2_(NCS)_2_ plus PANI composite tend to exhibit antiferromagnetic coupling between the nanoparticles and Fe(phen)_2_(NCS)_2_ when the latter is in the high spin state. The quenching of the XMCD signal with as much as 10% (loading by weight) of the Fe_3_O_4_ nanoparticles in the Fe(phen)_2_(NCS)_2_ plus PANI composite suggests that a considerable volume of Fe(phen)_2_(NCS)_2_ in the PANI is antiferromagnetically coupled to the Fe_3_O_4_.

## Data Availability

The Data will gladly be made available upon request.

## Supplementary Materials

The following supporting information can be downloaded at https://www.mdpi.com/article/10.3390/molecules29194574/s1, Figure S1: The Fourier transform IR (FTIR) spectrum of Fe(phen)_2_(NCS)_2_ nano-powder that was used for all bi-composite and tri-composite fabrication; Table S1: The hysteresis width in χ_M_T versus T plot for composites of Fe(phen)_2_(NCS)_2_ plus PANI plus varying concentrations of Fe_3_O_4_ (from 0.5 % up to 20 % by weight). The applied magnetic field is 2 T. Third column δ, is evaluated as the hysteresis width changes of that sample with respect to the hysteresis width of pristine Fe(phen)_2_(NCS)_2_, as the reference; Figure S2: The plots of χ_M_T versus T for composites of Fe(phen)_2_(NCS)_2_ plus PANI plus various concentrations of Fe_3_O_4_ nanoparticles (% loading by weight) with an external applied magnetic field of 2 T; Figure S3: Plots of χ_M_T versus T, for composites of Fe(phen)_2_(NCS)_2_ plus PANI plus varying concentrations of Fe_3_O_4_ (% by weight). The χ_M_T versus T of (a) pristine Fe(phen)_2_(NCS)_2_ crystallites while Fe(phen)_2_(NCS)_2_ plus PANI is plotted in black, (b) Fe(phen)_2_(NCS)_2_ plus PANI plus 5% (by weight) of Fe_3_O_4_, (c) [Fe(phen)_2_(NCS)_2_] plus PANI plus 1% (by weight) of Fe_3_O_4_, (d) [Fe(phen)_2_(NCS)_2_] plus PANI plus 10% (by weight) of Fe_3_O_4_. The applied magnetic field is 2 T; Figure S4: The χ_M_T versus T plot for (gray) Fe(phen)_2_(NCS)_2_ and (red) Fe(phen)_2_(NCS)_2_ plus PANI composite. The applied magnetic field is 2 T; Figure S5: The XAS spectra of Fe(phen)_2_(NCS)_2_ plus PANI plus and Fe_3_O_4_ nanoparticles (5% loading by weight) composite at 80 K; Figure S6: The XAS spectra of Fe(phen)_2_(NCS)_2_ plus PANI plus and Fe_3_O_4_ nanoparticles (10% loading by weight) composite at 80 K; Figure S7: The XAS spectra of Fe(phen)_2_(NCS)_2_ plus PANI plus and Fe_3_O_4_ nanoparticles (5% loading by weight) composite at 300 K; Figure S8: The XAS spectra of Fe(phen)_2_(NCS)_2_ plus PANI plus and Fe_3_O_4_ nanoparticles (10% loading by weight) composite at 300 K.
