# Supplementary material for: Evidence of Cooperative Effects for the Fe(phen)2(NCS)2 Spin Crossover Molecular Complex in Polyaniline Plus Iron Magnetite
Source: Molecules. 2024 Sep 26;29(19):4574. doi: 10.3390/molecules29194574 (PMC11477724; doi:10.3390/molecules29194574)
Supplement: Supplementary file 1 [file molecules-29-04574-s001.zip › molecules-3185486-supplementary.pdf]

Supplementary materials:

Evidence of Cooperative Effects for the Fe(phen)2(NCS)2 Spin Crossover Molecular Complex in Polyaniline plus Iron Magnetite

Wai Kiat Chin [1,†] , Binny Tamang [2,†], M. Zaid Zaz [1], Arjun Subedi [1], Gauthami Viswan [1] , Alpha T. N'Diaye [3], Rebecca Y. Lai [2,*] and Peter A. Dowben [1,*]

1 Department of Physics and Astronomy, University of Nebraska-Lincoln, Lincoln, NE 68588, USA; wchin6@huskers.unl.edu (W.K.C.); zzaz2@huskers.unl.edu (M.Z.Z.); arjun.subedi@huskers.unl.edu (A.S.); gviswan2@huskers.unl.edu (G.V.)
2 Department of Chemistry, University of Nebraska-Lincoln, Lincoln, NE 68588, USA; btamang2@huskers.unl.edu
3 Advanced Light Source, Lawrence Berkeley National Laboratory, Berkeley, CA 94720, USA; atndiaye@lbl.gov
* Correspondence: rlai2@unl.edu (R.Y.L.); pdowben1@unl.edu (P.A.D.)
† These authors contributed equally to this work.

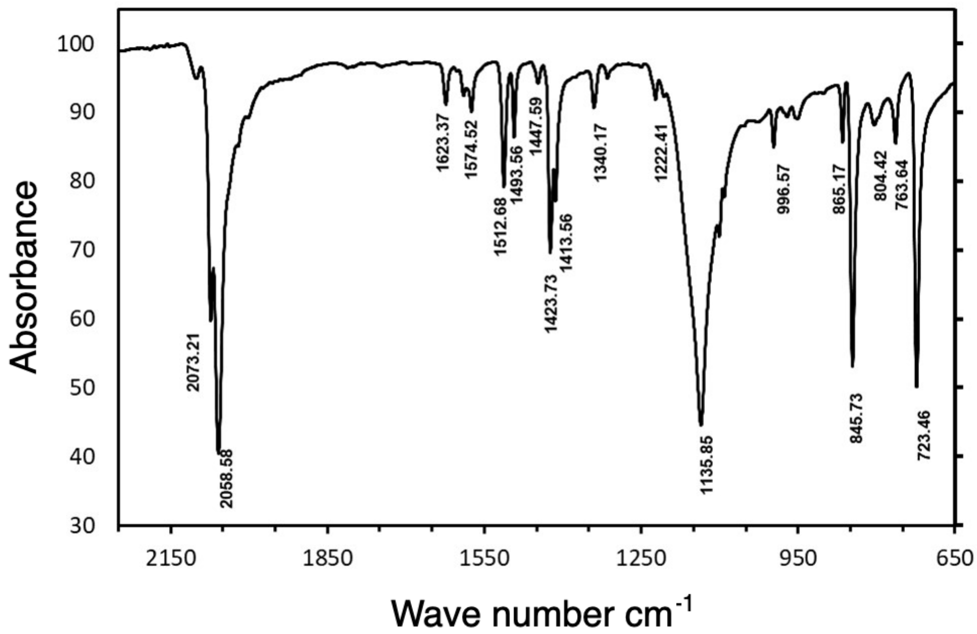

Figure S1. The Fourier transform IR (FTIR) spectrum of $Fe(phen)_2(NCS)_2$ nano-powder that was used for all bi-composite and tri-composite fabrication.

Composites	ΔT (K)	δ
pristine $Fe(phen)_2(NCS)_2$	10.6	$N.a$
$Fe(phen)_2(NCS)_2$ plus PANI	11.3	0.7
$Fe(phen)_2(NCS)_2$ plus PANI plus Fe_3O_4 (0.5 % by weight)	10.7	0.1
$Fe(phen)_2(NCS)_2$ plus PANI plus Fe_3O_4 (1.0 % by weight)	10.5	0.1
$Fe(phen)_2(NCS)_2$ plus PANI plus Fe_3O_4 (2.0 % by weight)	10.1	0.5
$Fe(phen)_2(NCS)_2$ plus PANI plus Fe_3O_4 (3.0 % by weight)	9.2	1.4
$Fe(phen)_2(NCS)_2$ plus PANI plus Fe_3O_4 (4.0 % by weight)	8.8	1.8
$Fe(phen)_2(NCS)_2$ plus PANI plus Fe_3O_4 (5.0 % by weight)	9.9	0.7
$Fe(phen)_2(NCS)_2$ plus PANI plus Fe_3O_4 (6.0 % by weight)	8.6	2
$Fe(phen)_2(NCS)_2$ plus PANI plus Fe_3O_4 (7.0 % by weight)	9.5	1.1
$Fe(phen)_2(NCS)_2$ plus PANI plus Fe_3O_4 (8.0 % by weight)	9.5	1.1
$Fe(phen)_2(NCS)_2$ plus PANI plus Fe_3O_4 (9.0 % by weight)	9.3	1.3
$Fe(phen)_2(NCS)_2$ plus PANI plus Fe_3O_4 (10.0 % by weight)	9.5	1.1
$Fe(phen)_2(NCS)_2$ plus PANI plus Fe_3O_4 (15.0 % by weight)	9.2	1.4
$Fe(phen)_2(NCS)_2$ plus PANI plus Fe_3O_4 (20.0 % by weight)	9.7	0.9

Table S1. The hysteresis width in $\chi_M T$ versus T plot for composites of $Fe(phen)_2(NCS)_2$ plus PANI plus varying concentrations of Fe_3O_4 (from 0.5 % up to 20 % by weight). The applied magnetic field is 2T. Third column δ, is evaluated as the hysteresis width changes of that sample with respect to the hysteresis width of $Fe(phen)_2(NCS)_2$ alone, as the reference.

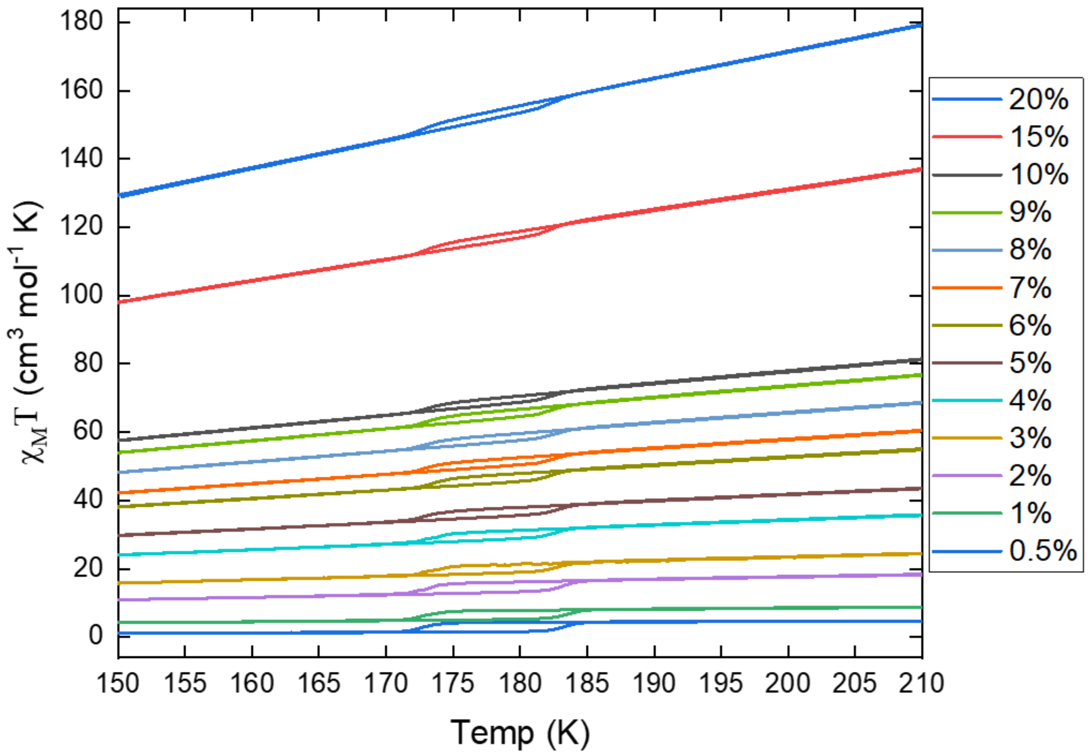

Figure S2. The plots of $\chi_M T$ versus T for composites of $Fe(phen)_2(NCS)_2$ plus PANI plus various concentrations of Fe_3O_4 nanoparticles (% loading by weight) with an external applied magnetic field of 2 T.

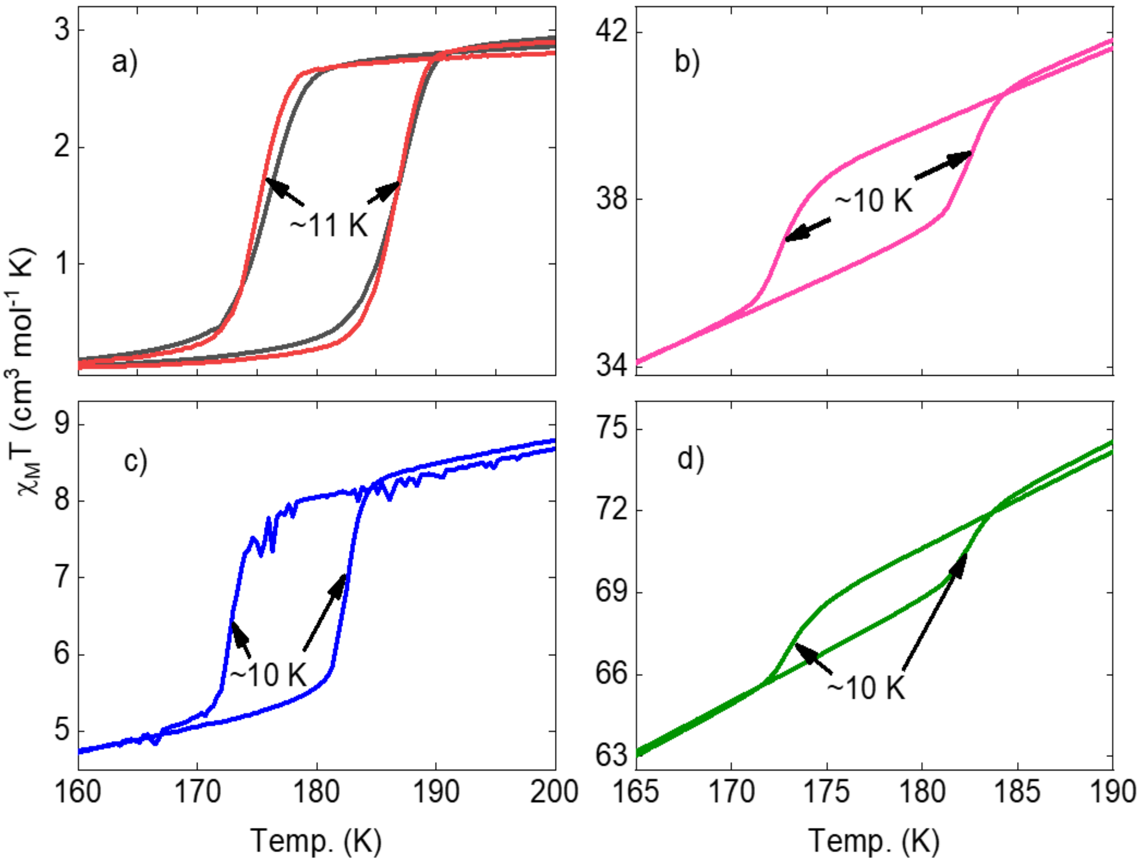

Figure S3. Plots of $\chi_M T$ versus T, for composites of $Fe(phen)_2(NCS)_2$ plus PANI plus varying concentrations of Fe_3O_4 (% by weight). The $\chi_M T$ versus T of (a) pristine $Fe(phen)_2(NCS)_2$ crystallites while $Fe(phen)_2(NCS)_2$ plus PANI is plotted in black, (b) $Fe(phen)_2(NCS)_2$ plus PANI plus 5% (by weight) of Fe_3O_4, (c) $Fe(phen)_2(NCS)_2$ plus PANI plus 1% (by weight) of Fe_3O_4, (d) $Fe(phen)_2(NCS)_2$ plus PANI plus 10% (by weight) of Fe_3O_4. The applied magnetic field is 2 T.

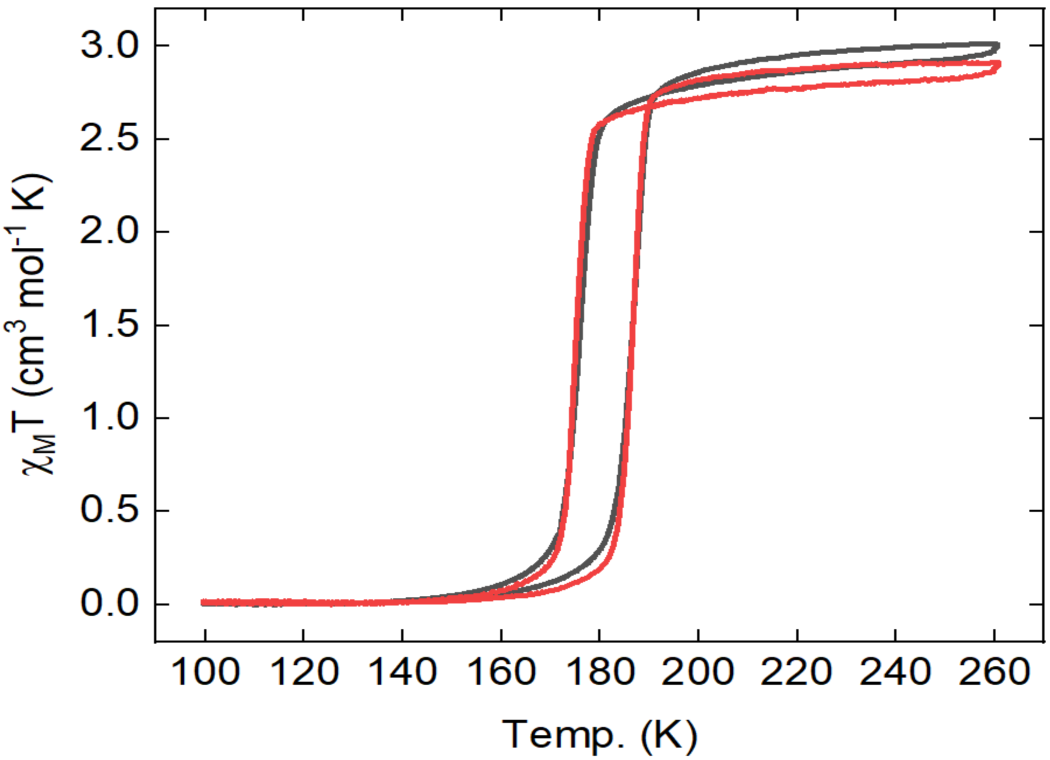

Figure S4. The $\chi_M T$ versus T plot for (gray) $Fe(phen)_2(NCS)_2$ and (red) $Fe(phen)_2(NCS)_2$ plus PANI composite. The applied magnetic field is 2 T.

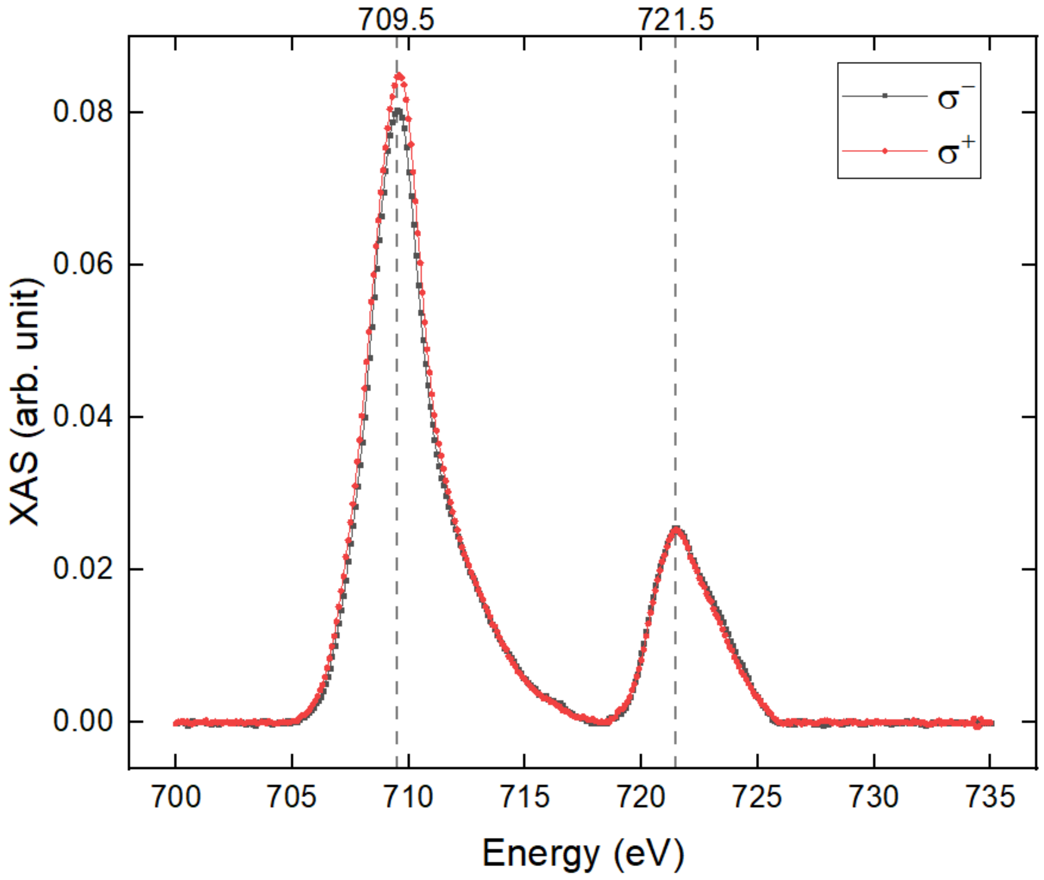

Figure S5. The XAS spectra of $Fe(phen)_2(NCS)_2$ plus PANI plus and Fe_3O_4 nanoparticles (5% loading by weight) composite at 80 K.

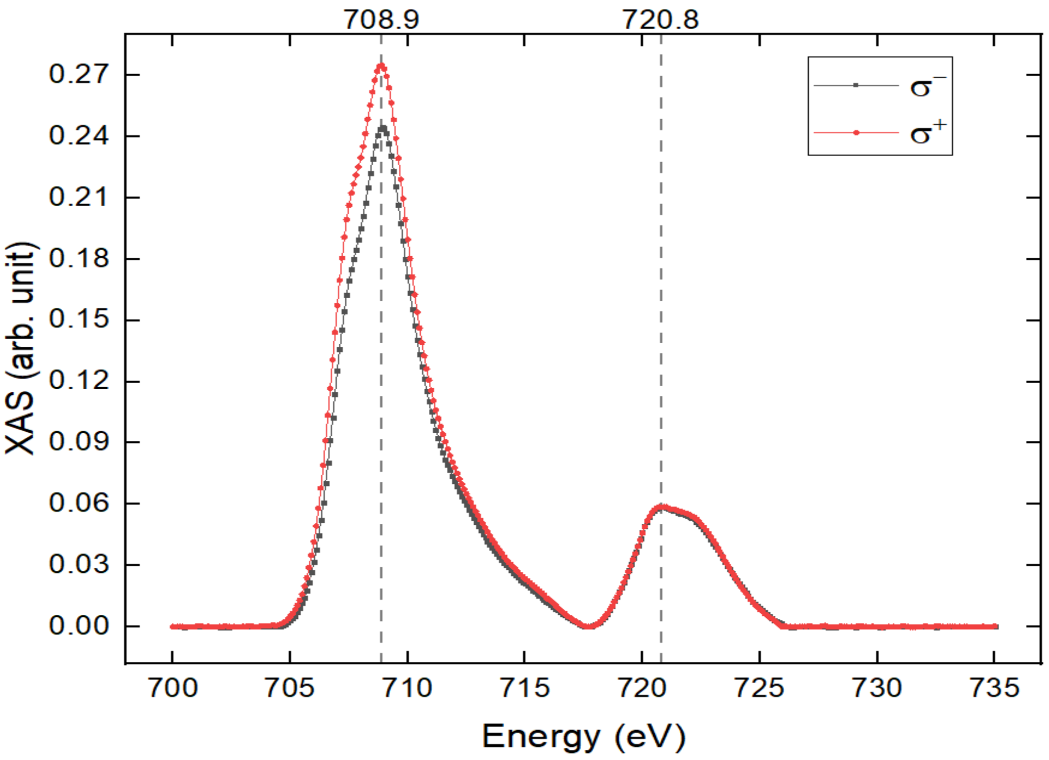

Figure S6. The XAS spectra of $Fe(phen)_2(NCS)_2$ plus PANI plus and Fe_3O_4 nanoparticles (10% loading by weight composite at 80 K.

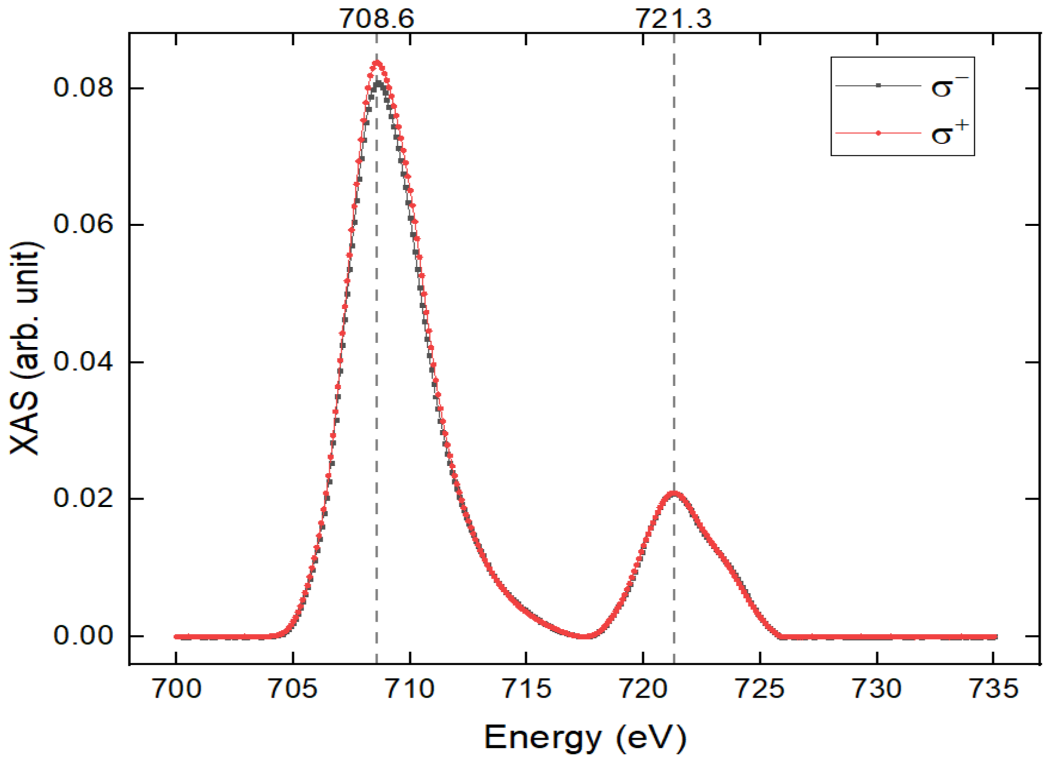

Figure S7. The XAS spectra of $Fe(phen)_2(NCS)_2$ plus PANI plus and Fe_3O_4 nanoparticles (5% loading by weight) composite at 300 K.

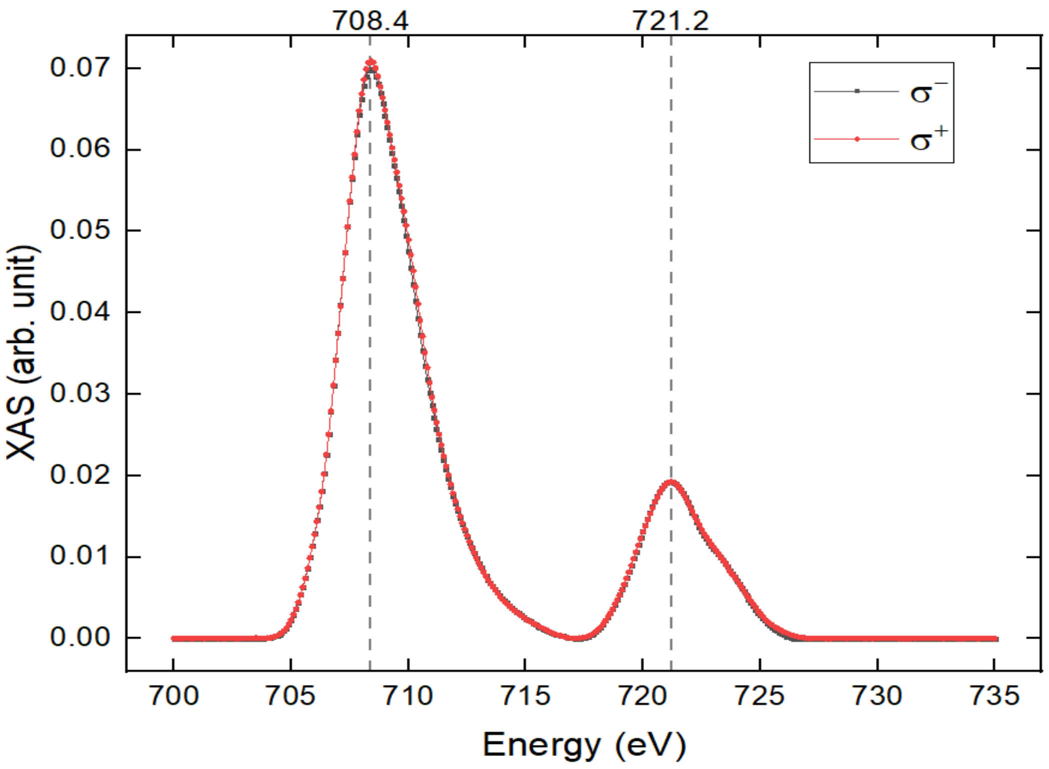

Figure S8. The XAS spectra of $Fe(phen)_2(NCS)_2$ plus PANI plus and Fe_3O_4 nanoparticles (10% loading by weight) composite at 300 K.

Acknowledgements

This work was supported by the National Science Foundation (NSF) through the NSF-DMR-EPM 2317464 (WKC, MZZ, GV, and PAD), EPSCoR RII Track-1: Emergent Quantum Materials and Technologies (EQUATE), award number OIA-2044049 (AS, RL) and NSF-PREM award number 1827690 (AS, RL and BT). LBNL was supported by the Office of Science, Office of Basic Energy Sciences, of the US DOE (Contract No. DE-AC02-05CH11231).
